# View-Based Organization and Interplay of Spatial Working and Long-Term Memories

**DOI:** 10.1371/journal.pone.0112793

**Published:** 2014-11-19

**Authors:** Wolfgang G. Röhrich, Gregor Hardiess, Hanspeter A. Mallot

**Affiliations:** Cognitive Neuroscience Unit, Department of Biology, University of Tübingen, Tübingen, Germany; University of Sussex, United Kingdom

## Abstract

Space perception provides egocentric, oriented views of the environment from which working and long-term memories are constructed. “Allocentric” (i.e. position-independent) long-term memories may be organized as graphs of recognized places or views but the interaction of such cognitive graphs with egocentric working memories is unclear. Here we present a simple coherent model of view-based working and long-term memories, together with supporting evidence from behavioral experiments. The model predicts 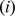 that within a given place, memories for some views may be more salient than others, 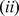 that imagery of a target square should depend on the location where the recall takes place, and 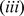 that recall favors views of the target square that would be obtained when approaching it from the current recall location. In two separate experiments in an outdoor urban environment, pedestrians were approached at various interview locations and asked to draw sketch maps of one of two well-known squares. Orientations of the sketch map productions depended significantly on distance and direction of the interview location from the target square, i.e. different views were recalled at different locations. Further analysis showed that location-dependent recall is related to the respective approach direction when imagining a walk from the interview location to the target square. The results are consistent with a view-based model of spatial long-term and working memories and their interplay.

## Introduction

As we walk through an environment, we constantly keep track of objects, landmarks, and path opportunities around us. This environmental information forms a working memory of surrounding space for which Loomis, Klatzky, and Giudice [1] suggested the term “spatial image”. Local, ego-centric representations of space have been studied in many contexts, including among others sensori-motor integration, visual scene recognition, and spatial cognition. Tatler and Land [2] and Land [3] review a large body of evidence on ego-centric visual representation supporting the stability of perception across eye-movements as well as eye-hand coordination with and without locomotion of the body. The representation considered by Tatler and Land [2] extends around the agent up to about the size of a room in an indoor environment. A similar spatial working memory including also a mechanism for spatial updating has been suggested by Byrne, Becker, and Burgess [4]. The notion of the spatial image [1] is slightly more general in that it may include knowledge from other (non-visual) modalities and extends to more distant spaces, which may be out of sight even if the observer would turn his or her head accordingly. Information from distant locations beyond the current sensory horizon can originate from two sources, i.e. long-term memory of distant places, or spatial updating if the distant place had been visited before and was since maintained in working memory.

### Multiple representations of space

Multiple representations of space have been suggested for a number of reasons. One issue is the problem of scale which may vary from centimeters in manipulation tasks to thousands of kilometers in way-finding. Grüsser [5] distinguishes a (mostly metrical) grasp space, a near- and a far-distance action space, and a visual background. Montello [6] presented a classification of “psychological spaces” also based on scale, in which the spatial image discussed here is somewhere between “vista space” (what is currently visible) and “environmental space” (the area a subject is used to navigate in).

The distinction between working and long-term memories of space is grounded both in behavioral and neurophysiological data [7], [8]. Spatial working memory tasks which are largely independent of spatial long-term memories include spatial sequence learning such as walking versions of the Corsi block-tabbing task [9], perspective taking and spatial updating [10], walking without vision [11], path integration [12], [13], path-planning in multi-local tasks [14], etc. Interactions of spatial working and long-term memories are crucial in way-finding, i.e. the planning of novel paths from known segments [15]–[17], spatial imagery [18], direction giving, and other tasks. Wang and Brockmole [19] studied spatial updating, a typical working memory task, in nested environments and concluded that spatial updating acts differently on close (the surrounding room) and distant (the outdoor buildings) environments. Giudice, Klatzky, Bennett, and Loomis [20] addressed the interaction of long-term and working memories in a pointing task involving the angle between items stored in the different memory systems.

In a study by Basten, Meilinger, and Mallot [21], visitors of the University restaurant of the University of Tübingen were asked to draw sketches of the “Holzmarkt”, a central and familiar downtown square about two kilometers away. Drawings were rated for orientation and a clear preference for the southward view was found, depicting a landmark church building on top of a hill. However, when subjects had been asked prior to the sketching task to imagine walking a route passing by the target square in one of two opposite directions, drawings in the respective viewing direction became significantly more frequent. The authors concluded that mental travel activated a view-dependent (“ego”-centric with respect to the imagined travel) representation of the target square which later primed the sketching process.

A particularly interesting case for the present discussion is representational neglect studied by Bisiach and Luzzatti [22], which shows that (at least in patients suffering from hemilateral neglect), recall of spatial long-term memories depends on the subject's imagined position and orientation. One obvious interpretation of this finding is that recall from long-term memory goes into some sort of spatial image, or working memory centered at the observer's imagined position and that it is the left side of this representation which is affected by neglect.

Spatial memory systems may differ in the reference system employed to organize spatial information. Perception is ego-centric and so is the assumed spatial image [1], [2], [23]. In perspective taking, route planning, and mental travel, ego-centric memories centered at imagined positions may also exist. The reciprocal term, allocentric, is harder to define. Summarizing discussions e.g. by Klatzky [24], Burgess [23], and Mallot and Basten [25], we define an allocentric memory as one that does not change as the observer moves. Note that this definition does not refer to coordinate systems or global anchor points. Indeed knowledge such as distances between places as well as oriented views and their relation to other oriented views qualifies as allocentric memory in this sense, because it can be carried around and remains useful without a need for movement-dependent changes or transformations. Almost as a corollary to this definition, long-term memories will always be allocentric, while working memories involving automated spatial updating will be not. In the Model section, we describe the view-graph [26] as an allocentric data structure for spatial long-term memory that lends itself easily to interactions with ego-centric working memories.

Over the past decade, imaging studies have identified an extensive network of cortical and subcortical brain areas involved in a variety of spatial behaviors. Tasks involving an interplay of spatial long-term and working memories have been shown to recruit structures such as the retrosplenial cortex as well as medial temporal lobe [27]–[30]. More on the visual side, scene recognition as well as imagery of out-of-sight places or perspectives has been related to various parts of the parietal cortex and transverse occipital sulcus [31]–[33].

### A view-based model of spatial working and long-term memories

In the interplay between spatial working and long-term memories, the encoding, or data-format, used by each memory structure is of great importance. Recall from long-term memory into spatial working memory, i.e. between allocentric and ego-centric representations, is often thought to require a coordinate transform, which is certainly true if spatial information is explicitly represented in the form of coordinates. However, in a view-based account, an allocentric, long-term representation of place may even be a view or a collection of views which were egocentric when first perceived and stored, but are now carried around for reference. Simply enough, transformation of this view-based allocentric representation into an egocentric one amounts to picking a particular view which corresponds to the current viewing direction and loading this view into working memory, e.g. for comparison to the currently visible view of the present place. As a result, places would be recognized by view matching [34], similar to the snapshot algorithms discussed in insects [35]. In addition to simple matching, a process of view transformation might be involved, allowing the prediction of nearby or intermediate views from stored ones, as has been suggested for robot applications [36]. Such a mechanism seems to be required also in the pointing task studied by Giudice et al. [20], involving both long-term and working memories. In pose-invariant object recognition, view interpolation is a well-established mechanism [37], [38].

The concept of view-based representations of navigational space has been developed by Schölkopf and Mallot [26] and used in robot simulations [39] and models of hippocampal processing [40]. Behavioral evidence for view-based navigation in humans has been presented by [41]–[43]. View specific neuronal activity has been reported e.g. from the monkey parahippocampal formation [44] or the human retrosplenial cortex [30].

**Figure 1:**
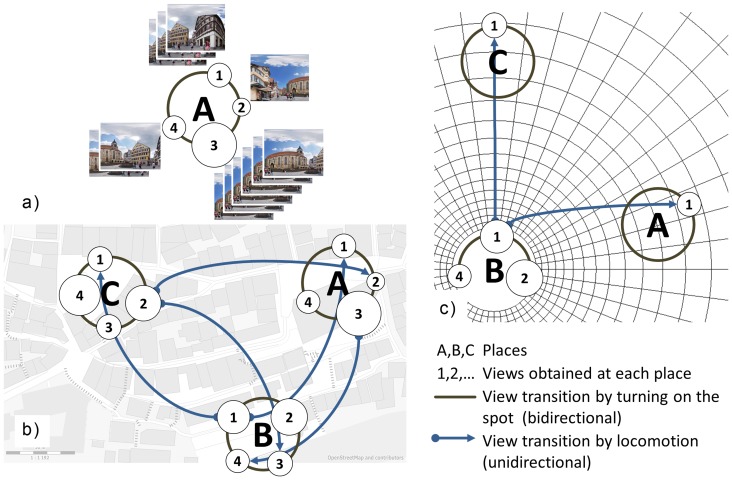
View-based model of spatial long-term memory. Upper case letters A, B, C denote places, numbers (1–4) denote views visible at each place. E.g., view A3 depicts a church building when standing at the “Holzmarkt” (A), facing south. a) Place representation composed of a collection of directional views (1–4) obtained at a place A. Views may be represented multiply, or overlapping, allowing to represent viewing direction in a population code. The size of the circles indicates the frequency with which each view is stored, or the likelihood that it is read out in recall. (Tübingen Holzmarkt icons are sections of a panoramic image retrieved with permission from www.kubische-panoramen.de.) b) View-graph of 12 views (A1-C4) belonging to three places. Within each place, views are linked by turning movements. Views of different places are linked by movements involving translations. Note that these links are unidirectional; for example a path from A to B starts from view A3, while the return from B to A will end on A1. c) A view-based model of spatial working memory is obtained by extracting a sub-graph from the total view-graph. It contains the current view (B1) which also marks the current observer position and forward direction, and its outward neighborhood of order 1, i.e., the directly adjacent views (A1, B2, B4, C1). Outward neighborhoods of higher orders may also be represented but are not shown in the figure. The polar grid is added to indicate that metric updating may take place in the working memory, which, however, does not play a role in the experiment reported in this paper. Map source: © OpenStreetMap contributors.

The central spatial concept of the view-based framework is the view, i.e. an image or early visual representation of a sector or angle of the environment taken at a position 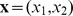 and with a viewing direction 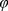; we denote the view by 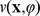. It need not be limited by the visual perimeter, but may also contain information from beyond the current visual horizon, encoded in an egocentric way, see, for example, Tatler and Land [2]. The simplest long-term memory of a place 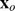 is then a collection of views taken at that place, 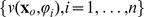 where the index 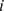 enumerates the individual viewing directions and 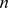 is the total number of views stored for the particular place (see Figure 1a). The views may be overlapping and the distribution of viewing directions 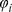 may be anisotropic. If, for example, one particular view of a place is especially salient, we may model this by assuming that multiple copies of this view, or largely overlapping adjacent views, will be included in the place representation. In analogy to object representation, such views might be called “canonical” for the respective place. In addition to the views themselves, we assume that the adjacencies of views are also represented in the place code. The views together with their adjacencies thus form a simple view-graph with a ring-topology. As in [26], the adjacency links will be labelled with action codes such as “turn left”, or “turn right 40 degrees”.

From this place representation, a long-term memory of a larger environment, i.e. a cognitive map, can be built as a full view-graph and used for way-finding (see Figure 1b). For multiple places, interplace view adjacencies have to be stored as “action labels” representing egocentric locomotor actions such as “walk straight from here” or “follow the street from here”. In these action labels, “here” refers to a view from the current place assuming the observer's current heading. The link will end at a view of a neighboring place, as it appears when arriving from the starting location. As was demonstrated by Schölkopf and Mallot [26], the resulting view graph contains sufficient information for route planning between connected views.

As a model of spatial working memory, we suggest a sub-graph of the full view-graph, consisting of the current view corresponding to the observer's current position and orientation, and the views reachable from this current view in a small number of steps 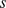, i.e. the outward neighborhood 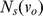. Note that the graph links are directed, allowing to distinguish an outward neighborhood (views reachable from 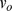) from an inward neighborhood (views from which 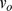 can be reached). In Figure 1c, we show the one-step (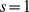) outward neighborhood of view 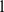 of place B. As the observer moves, the current view will change and so will its outward neighborhood represented in working memory. This may be achieved by repeatedly refreshing the neighborhood from long-term memory, i.e. loading the appropriate sub-graph after each movement step. Alternatively, or on smaller scales, one could think of some sort of ego-motion driven image transformation (spatial updating) within working memory. We indicate this possibility by adding a polar coordinate grid to working memory in Figure 1c. In our experiment, we cannot distinguish between refreshing from long-term memory and spatial updating within working memory. See [20] for an experiment directly addressing this problem.

When asked to imagine a nearby target place 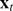, subjects will recall from memory one of the stored views 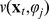 of this place. In spatial working memory, only the views contained in the outward neighborhood of 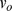 will be present. Therefore, if recall is based on working memory content, the view obtained when (mentally) traveling from the current “here” to the target place will be selected. In this case, we predict that in visual recall of a target place, the recalled viewing direction will depend on interview location. If, however, recall is based solely on long-term memory, one of the known views of the target place will be selected independent of interview location.

For the analysis of the data presented below, we introduce the following notation: Let 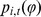 denote the probability that the recalled view of target place 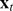 has the orientation 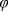, given that the interview location is 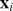. Let further 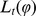 and 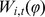 denote the probability densities of recalling a view 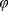 if recall is from long-term or working memory, respectively. Note that the working memory contribution depends on interview location, whereas the long-term memory contribution does not. We expect that 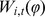 is a peaked distribution with a maximum at the approach direction from interview location 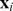 to target place 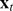. In the data analysis, we will identify the approach direction with the air-line direction between the two places, 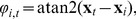
(1)where 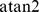 is the inverse tangent function with two arguments. For the distribution of the recalled view orientations, we obtain 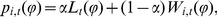
(2)where 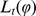 and 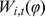 and are the long-term and working memory contributions, respectively and 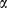 is a mixing factor varying between 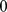 and 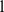. It reflects the relative strength of long-term and working memory components in the recall. We expect that 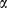 is less than 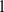 for interview sites close to the target place and 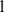 for distant interview locations.

If, for a given target place, the interview locations are spaced regularly around this place, the average of the 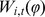 will approach the uniform distribution, 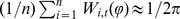 and we may estimate the long-term memory contributions as 
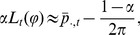
(3)where 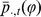 denotes the average view distribution over all interview locations. From this, we will calculate an estimate for the working memory contribution as 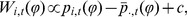
(4)where 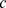 is a constant reflecting the non-zero average of the working memory distributions. In the analysis of the experimental data, orientations are sampled to the four cardinal directions (N, E, S, W). The constant 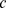 cancels out in the calculation of the circular vectors following Equation 5 below. In analyses of the distribution 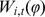 this constant is important to avoid negative values; it can be set to 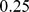. The proportionality factor in Equation 4 will be ignored in the sequel.

## Experiment 1 – “Holzmarkt”

### Material and Methods

**Figure 2:**
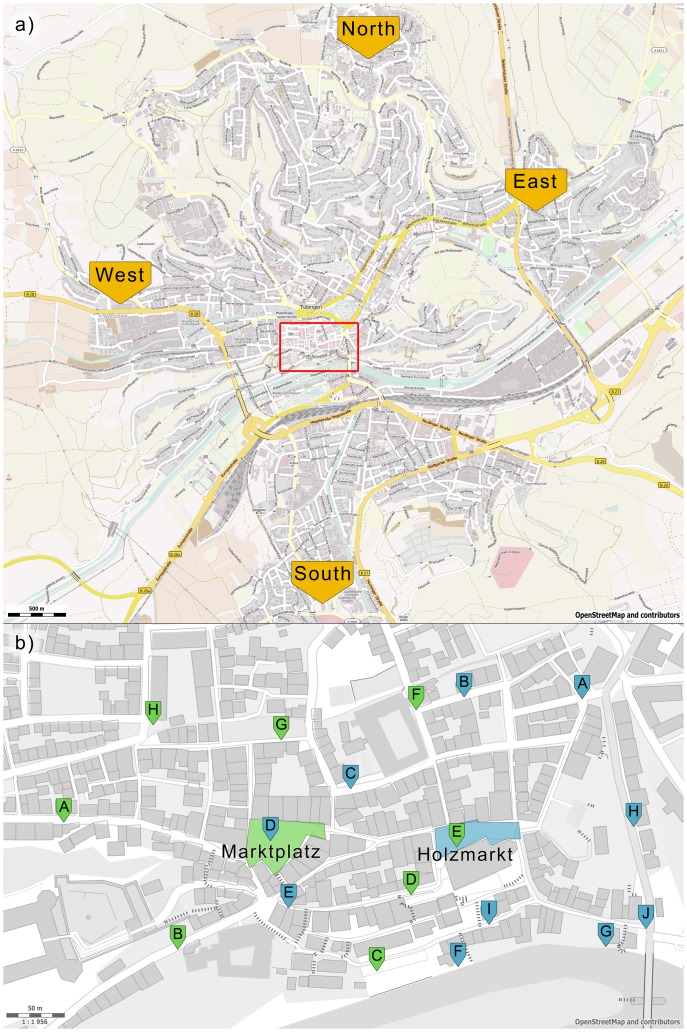
City maps of Tübingen with interview locations and target places (“Holzmarkt” & “Marktplatz”). a) Distant (suburban) interview locations (North, East, South, West) were located in small shopping areas about 2 km away from the target squares, which were inside the downtown area (red square). b) Close-up view of the downtown area of Tübingen. Blue: Interview locations (A–J) and target place for experiment 1 (“Holzmarkt”). Green: Interview locations (A–H) and target place for experiment 2 (“Marktplatz”). Map source: © OpenStreetMap contributors.

Passers-by at 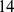 locations in Tübingen (see below and Figure 2) where approached during day time and asked “if they would participate in a quick interview for a navigational study”. They were informed about the type of the collected data and the general procedure. About one third agreed to participate (verbal informed consent) as was documented by their later participation in the interview. Participants were not asked for their names and accordingly were not required to give their consent in writing. Participants were free to terminate their participation at any time, simply by walking away. The informed consent procedures adheres to the guidelines of the Declaration of Helsinki, approval by the local ethics committee was not required.

**Figure 3:**
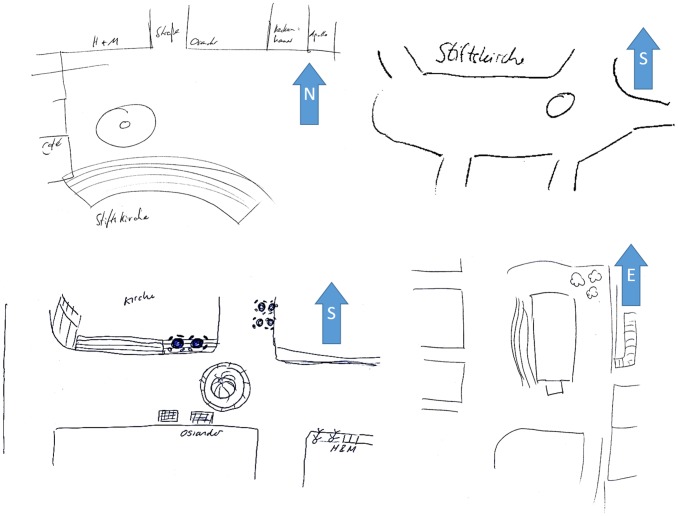
Examples of sketches of the “Holzmarkt” from four participants. The blue arrows indicate the orientation the sketch was rated in. Note the inscriptions “Stiftskirche” or “Kirche” referring to the landmark church building located at this square (see also view A3 in Figure 1a). The parallel lines mark a flight of stairs leading from the square to the church, the circles mark a fountain at the Western side of the square.

Participants were requested to “sketch the layout of the Holzmarkt” (timber market), a well-known down-town square, on an A4 sheet of paper. After sketching, they were asked for their age, years of residency in Tübingen, own judgment of general navigation skills, and own judgment of local knowledge (see below). Only sketches by subjects who had lived in Tübingen for more than two years were analyzed further. In total, these were 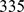 adults (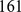 male, 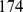 female). An interview and sketch map production took less than two minutes in total. Examples of sketch maps appear in Figure 3.

Interviews took place outdoors, either at one of four distant locations in small suburban shopping areas about 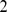 km away from the target square (“distant” condition) or at one of ten downtown locations in walking distance (about 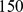 m) to but out of sight of the target square “Holzmarkt” (“near” condition; see Figure 3). Care was taken to approach participants walking in different directions. Approach was from sideways with respect to the participant's heading. Upon being approached, participants stopped but did not change their general body orientation. Also during recall, no regular turning movements of the participants were observed.

The sketches were categorized for orientation (North, East, South or West up) by three independent raters. From the 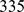 drawings 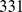 were judged identically (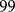
%) with a chance-corrected inter-rater reliability of 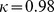. A small number of sketches was consistently rated diagonal; in these cases, the number 0.5 was added to the two adjacent directions. Only the 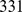 identically judged drawings were analyzed further (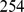 near condition, 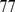 distant condition). The mean age of the 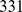 participants whose maps were included was 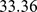 years, their average time of residency in Tübingen was 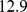 years, their own judgment of local knowledge and general navigation skills was 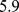 and 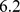, respectively, both on a scale between 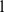 and 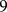 with 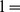 very poor and 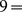 very good.

For each interview location 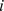, relative frequencies of ratings for the four cardinal directions were calculated and denoted as 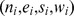 for North, East, South, and West. Average frequencies were also calculated separately of the ten “near” and the four “distant” interview locations and denoted as 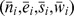. In the next step, the average frequencies from the “near” interview locations were subtracted from each of the local histograms of the “near” condition. Similarly, the average frequencies for the four distant interview locations were subtracted from the distant histograms. We refer to the results as the “location-dependent components” and consider them as an estimator of local working memory content, according to Equation 4. Finally, these location-depenedent components were transformed into location-dependent orientation vectors 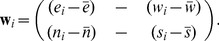
(5)The orientation of these vectors is an estimator of the circular mean of the working memory distribution 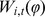 from Equation 4. The length is a measure of concentration of this distribution related to the circular variance [45], [46]. A long vector means more concentration (more coherent sketch orientations) and stronger differences from the average (long-term memory) distribution. Short vectors would result from sketch orientations that are similar to the long-term memory content.

### Results

**Figure 4:**
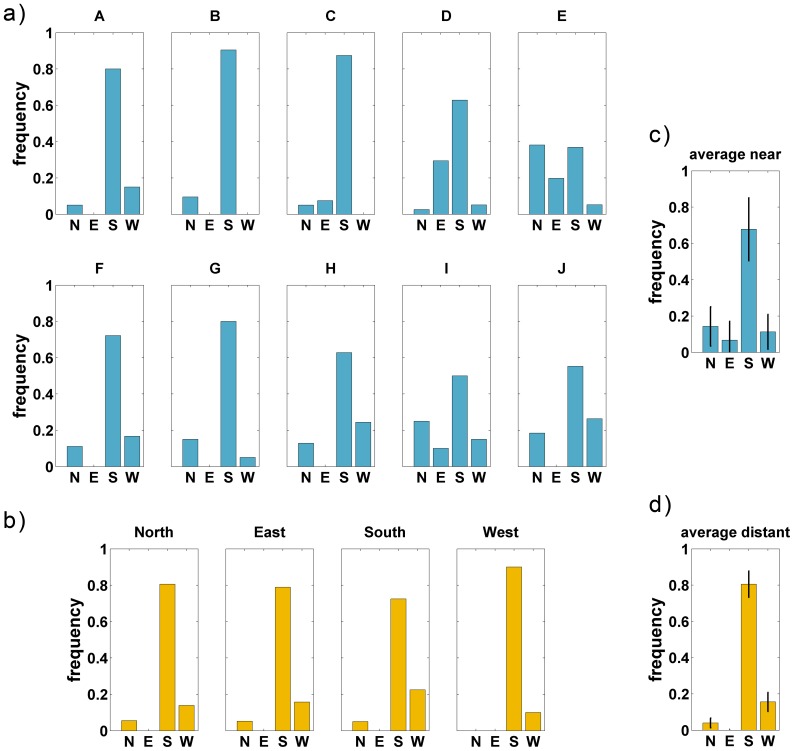
Sketch orientation frequencies for drawing the “Holzmarkt”. a) Orientation frequencies of the near interview locations (A–J). The obtained frequencies differed significantly from each other. b) Orientation frequencies of the distant interview locations (North to West). c, d) Average orientation frequencies with standard deviation of the near and distant condition, respectively. The 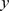-axis shows the frequency of sketch map orientations, the 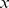-axis the rated orientation (North, East, South, West).

Orientation frequencies of the sketches of the ten downtown and four suburban interview locations are shown in Figure 4; for the orientation counts, see Figure S1. The distributions obtained at the near locations differ significantly from each other (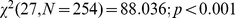) indicating that recalled view orientation depends on interview location. For the distant locations, no differences between the histograms could be found.

The average distributions for near and distant interview sites are shown separately in Figure 4. These distributions are significantly different from each other (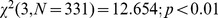) though comparable in shape.

**Figure 5:**
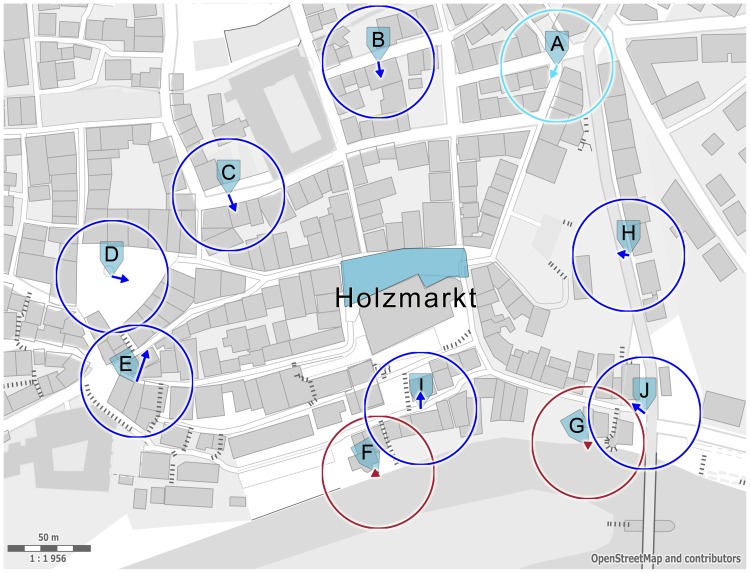
Downtown map of Tübingen with interview locations (A–J) for the near condition and target square “Holzmarkt”. The vectors show the average sketch map orientation at the respective interview site. At seven (blue circles) out of ten near sites sketch orientations were found to point from the interview location in the direction of the target square (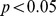 or better). At one location, a strong tendency was indicated (A, cyan, 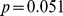). For two locations (F,G; red), no significant orientation effect could be found. Vector length ranges from zero to one (radius of circle) and is a measure of concentration of the location-dependent vectors. Map source: © Open-StreetMap contributors.

The orientation vectors obtained from the location-dependent components of the downtown interview locations (Equation 5) are plotted in Figure 5 superimposed on a map of Tübingen showing the target and interview locations. An overall tendency of the vectors to point to the target square is clearly apparent.

In order to test this tendency, we calculated the angular deviation between the location dependent orientation vectors and the theoretical air-line vector obtained for each interview location by subtracting the coordinates of the target square (defined as the center of gravity of the blue area in Figure 5) from the coordinates of the interview sites (Equation 1). For each interview location, the deviation or bias of the data from a uniform distribution towards the theoretical direction was tested with the circular 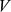-test [45], [46], taking into account the vector length as a measure of concentration. The deviations towards the theoretical direction are significant (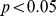 or better) for seven out of ten interview locations, and marginally significant for an additional one (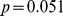). For two interview locations (F and G in Figure 5), no significant deviation from uniformity could be demonstrated.

**Figure 6:**
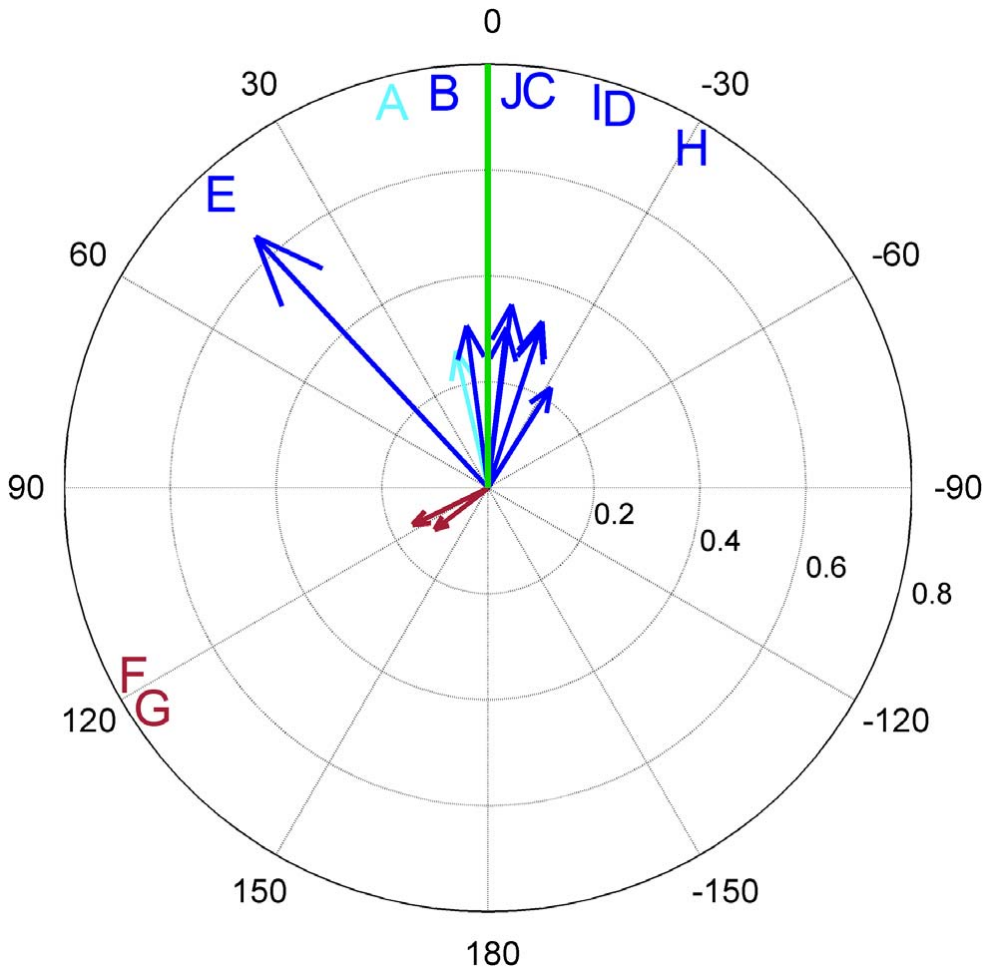
Location-dependent vectors from Fig. 5, rotated to align the air-line directions from all interview locations to 0 degrees (letters indicate interview locations). Vectors are significantly biased towards the theoretical direction (green line, 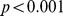). Vector length reaches from zero to one (radius of circle) and is a measure of concentration of the location-dependent vectors.

Figure 6 shows the location-dependent vectors rotated such that the theoretical direction for each interview location appears in upwards direction. For this sample of 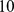 vectors, we again applied the circular 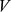-test, this time with the 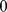-degree-vector as a theoretical direction. For the overall sample, bias towards the theoretical direction was significant with (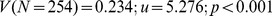).

For the four distant interview locations no such orientation effect could be found (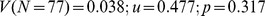).

## Experiment 2 – “Marktplatz”

To test the robustness of the findings of the first experiment with respect to other target squares, we chose another well-known square and repeated the previous experiment.

### Material and Methods

Eight new interview locations around the “Marktplatz” (market square) were selected for the near condition (Figure 2b, green). For the distant condition the same locations as in experiment 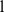 were used except for the southern one, which we did not again get access to. 330 passers-by agreed to participate. The procedure was the same as in experiment 1.

Sketches were again categorized for orientation (North, East, South or West up) by three independent raters. From the 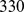 drawings 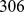 were judged identically (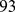
%) with a chance-corrected inter-rater reliability of 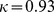. Only the 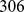 identically judged drawings were analyzed further (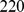 near condition, 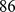 distant condition). The mean age of the 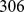 participants (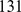 male, 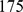 female) whose maps were included was 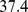 years, their average years of residency in Tübingen was 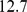, their own judgment of local knowledge was 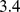 (with 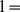 very poor and 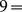 very good) and own judgment of how often they frequent the “Marktplatz” was 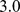, with 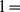 very rarely and 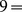 very often.

Average orientation frequencies for the near and distant conditions were calculated and subtracted from the histogram of the near and distant interview locations, respectively, yielding the location-dependent components of each distribution.

### Results

**Figure 7:**
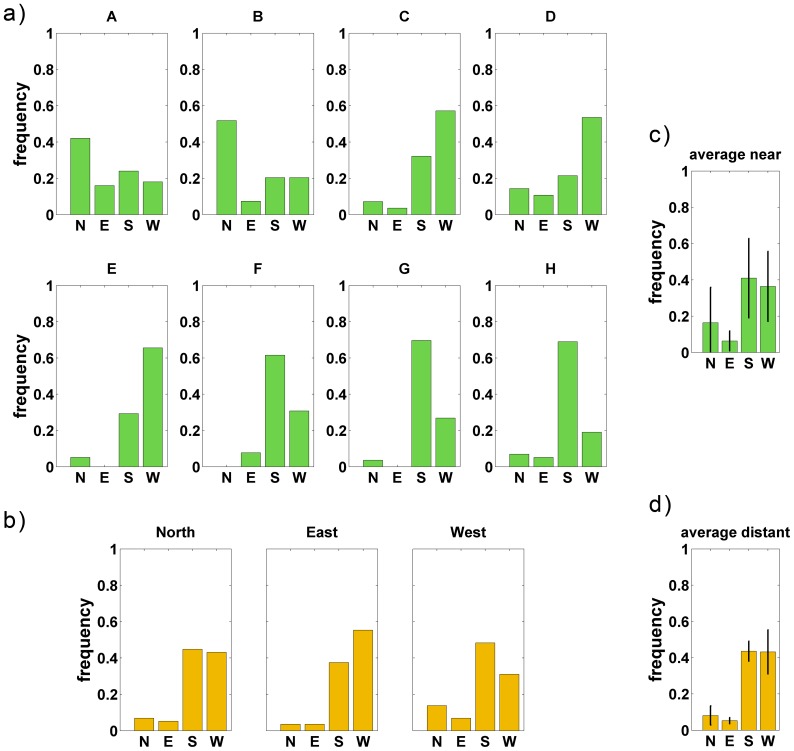
Sketch orientation frequencies for drawing the “Marktplatz”. a) Orientation frequencies of the near interview locations (A–H). The obtained frequencies differed significantly from each other. b) Orientation frequencies of the distant interview locations (North, East and West). No significant difference could be found. c, d) Average orientation frequencies with standard deviation of the near and distant condition, respectively. The 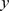-axis shows the frequency of sketch map orientations, the 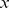-axis the rated orientation (North, East, South, West).

Orientation frequencies of the sketches of the eight near interview locations differed significantly from each other (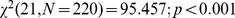). For the distant locations, no difference between the histograms could be found (Figure 7). Also, there was no significant difference between the near and distant average frequencies (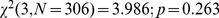).

**Figure 8:**
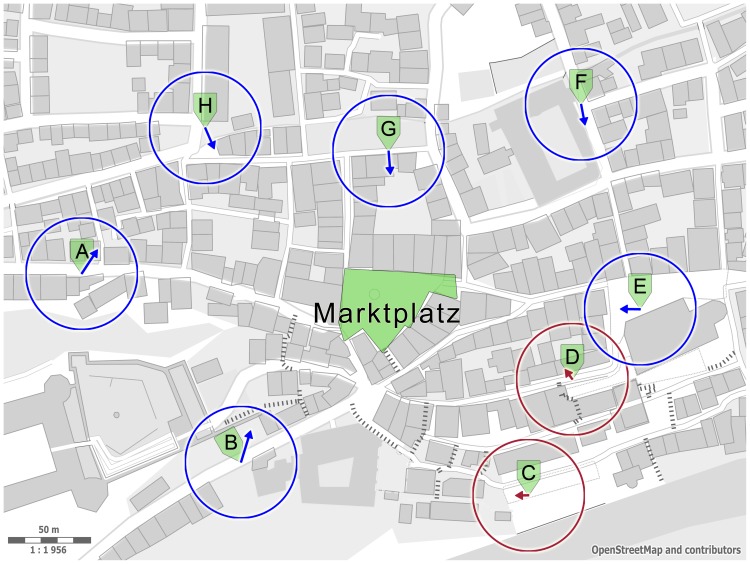
Downtown map of Tübingen with target square “Marktplatz”, near interview locations (A–H) and location-dependent vectors drawn at these locations. Vectors at six (blue circles) out of eight interview sites point towards the target square (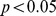 or better). For two locations (C, D; red), no significant orientation effect could be found. Vector length reaches from zero to one (radius of circle) and is a measure of concentration of the location-dependent vectors. Map source: © OpenStreetMap contributors.

**Figure 9:**
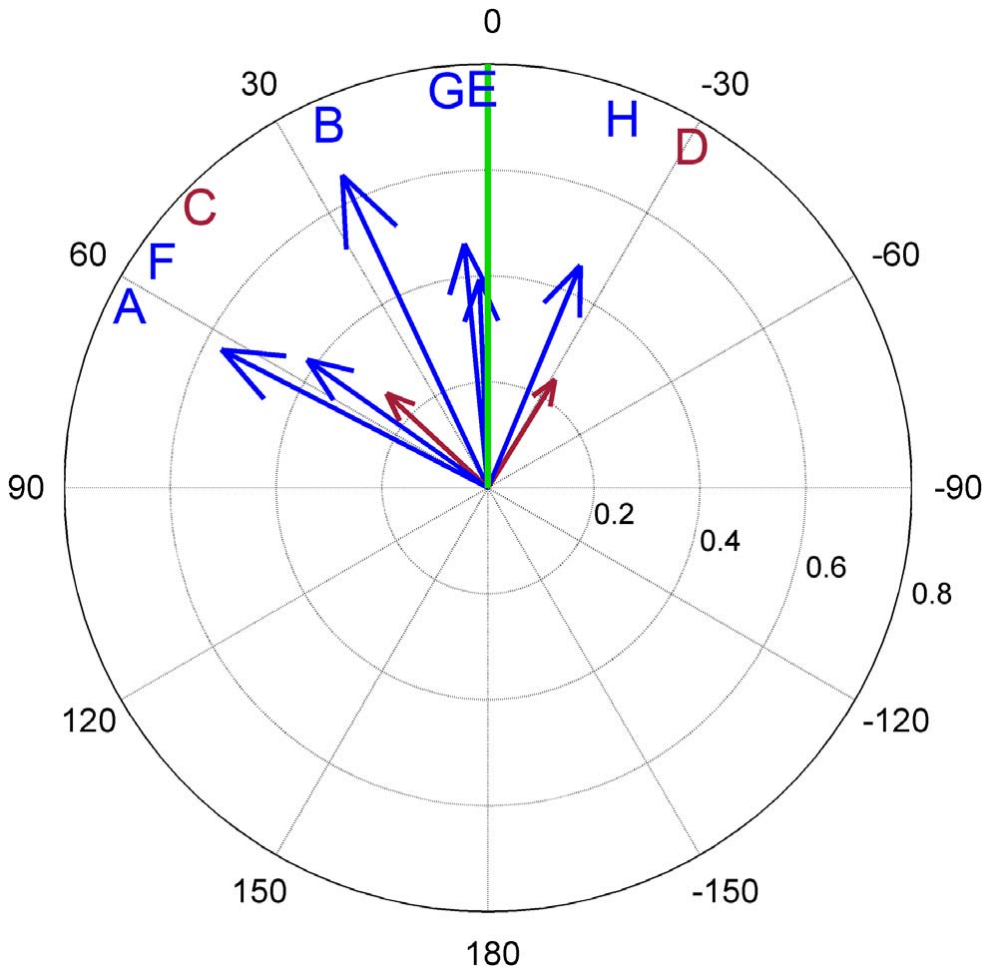
Location-dependent vectors from Fig. 8, rotated to align the air-line directions from all interview locations to 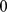 degrees (letters indicate interview location). Vectors are significantly biased towards the theoretical direction (green line, 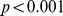). Vector length reaches from zero to one (radius of circle) and is a measure of concentration of the location-dependent vectors.

As shown in Figure 8, the majority of the location-dependent vectors of the near condition point towards the “Marktplatz” (center of gravity of green area in Figure 8). A significant bias of sketch orientations towards the air-line direction to the target square (center of gravity of the green area Figure 8) for six of the eight interview locations could be revealed by a circular 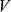-test. The sample of eight location-dependent vectors, rotated to align their respective air-line directions, also showed a highly significant bias towards the theoretical direction at zero degrees (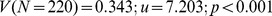) (Figure 9).

No bias could be detected for the three distant interview locations (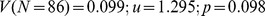).

## Discussion

The data presented in this study indicate that visuo-spatial recall of out-of-sight places does not occur with a random or fixed orientation but that recall orientation depends on both target and interview location.

The target square effect suggests a non-isotropic representation of each target square in long-term memory. For the distant (suburban) interview locations, orientation distributions were found that equal the average distributions taken over all near (downtown) locations. We therefore conclude that the target square dependence is underlying all our measurements and is modulated by interview location-dependent effects visible only for the downtown interview locations. The average view distribution for the “Holzmarkt” square (Exp. 1) is strongly peaked with a “canonical view” in southward direction, depicting a landmark church building on top of a hillock. In contrast, the view distribution for the “Marktplatz” (Exp. 2) is more isotropic, probably reflecting the more balanced salience of the surrounding houses. These differences are probably related to the specific topography of each place. The “Holzmarkt” is rising to the South, with a prominent church building on top. Approaches from behind the church (Northwards) are almost impossible and very rarely walked. Drawings with the church on top might thus be favored by familiarity, alignment with environmental axes and the fact that uphill buildings will appear on top of the sketching paper. In contrast, the salience of the buildings surrounding the “Marktplatz” (Exp. 2) is much more balanced. The “Marktplatz” is also rising to the South, but the most prominent building, the city hall, appears not on top but on the Western side. Also, approaches from all directions are possible and frequently walked. Still, a peak in the experimental data towards “South” and “West” is apparent here, too. We suggest that the long-term memory of either square is organized as a collection of discrete views (Figure 1a), sampling the various viewing directions with variable resolution much as has been suggested for view-dependence in face recognition [37]. Allocentric place memory might therefore be organized as a population code of orientation-specific memories. Indeed, neuronal specificities for views of places have been reported in the medial temporal lobe, see for example [47], [48].

The formation of one or several canonical views of a place requires further study, concerning potential relationships to canonical views of landmark objects and the selection of one view or another as canonical. Reasons for selection might include: Distinctiveness to other places, availability and distribution of local landmarks, geometric layout, visual salience of objects, path options and functionality, or intrinsic axes of the environment [49].

The distribution of recalled views depends also on interview location as was revealed by Chi-Squared tests on the orientation histograms. For the near (downtown) interview locations each local distribution is biased towards a preferred orientation roughly corresponding to the air-line direction from the interview location to the target square. A view of the target square, oriented in the current approach direction, thus seems to be activated in a spatial working memory either by automated spatial updating when walking in the city, or by a mental travel initiated when asked to draw the sketch, or by both effects (see Figure 1b,c). Spatial updating itself could again be achieved by two mechanisms, either image transformation as discussed in view-based object recognition [38] or by refreshing working memory from long-term memory.

In the introduction, we presented a view-based model of spatial recall predicting that the directional distributions of recalled sketch maps are a mixture of a fixed long-term memory distribution and a set of position dependent working memory distributions (Equation 3). As a direct test of this model, we performed a maximum likelihood analysis assuming for the orientation histograms a multinomial distribution with four possible outcomes (N, E, S, W) and theoretical probabilities 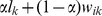, where 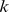 numbers the four possible outcomes and 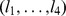 are the class averages over all interview locations, i.e. the assumed long-term memory contributions. The log likelihood function reads 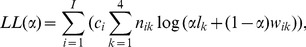
(6)where 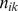 is the number of orientations 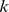 found at interview location 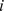 and the constant 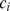 is the log of the multinomial coefficient for the local orientation distribution. Theoretical estimates for the working memory contributions at each interview location are derived from the local air-line directions 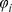 (Equation 1). The theoretical outcome probabilities for the assumed working memory distributions were set to 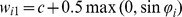, 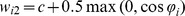, 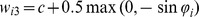, and 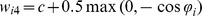, where 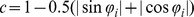 is a constant assuring that the four probabilities will add to 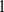. This distribution has the circular mean 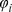 and variance 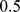, which reasonably approximates the location-dependent components shown in Figures 5 and 8.

**Figure 10:**
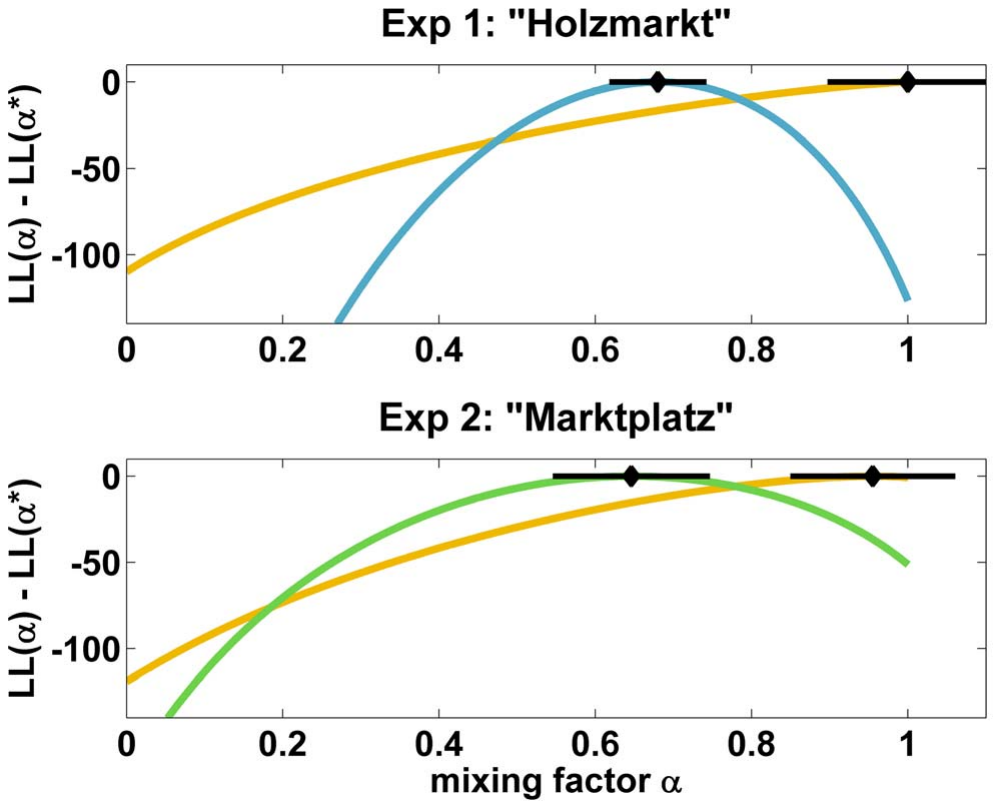
Likelihood analysis of the mixing model. Yellow: Distant locations, relative likelihood peaks for 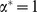, indicating that orientation distributions do not depend on air-line direction. Blue and green: Near locations, relative likelihood peaks at 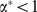, indicating the orientation distributions do depend on air-line direction in this condition. The black markers indicate with 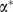 with 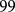
% confidence intervals. The 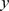-axis shows the relative log likelihood 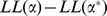, the 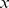-axis the mixing factor for working and long-term memory contributions.

Figure 10 shows the relative log likelihood 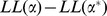 as a function of the mixing parameter 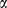 separately for the near and far interview locations in both experiments. For the “far” cases, the maximum likelihood estimator 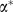 is 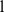, i.e. adding position-dependent working memory contributions to the model does not improve likelihood in these cases. In contrast, for the “near” cases, the maximum likelihood estimates lie between 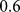 and 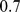; the horizontal lines in the plot are 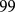
% confidence intervals. A likelihood ratio test for 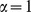 vs. 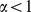 is significant with 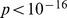 for the “near” cases in either experiment. The model with the location-dependent working memory component thus significantly improves the fit of the data.

We cannot decide from our data whether recall bias is strictly toward the air-line direction or toward the actual entry view obtained when walking to the target place along the street network, although in a view-based account, the latter seems more plausible. Indeed, this might have been the problem with the interview location D in experiment 2 from which two roughly equidistant routes to the target place exist, each with opposite entry directions into the target square.

No location-dependent effect was found for the distant (suburban) interview locations. We conclude that in these cases, recall did not depend on working memory processes such as spatial updating or mental travel. Of course, other working memory effects might still be involved. Since we used only two distance conditions, downtown and suburban, we cannot decide how far the location-dependent effect extends around the target place or if there is a gradual decay as could be modelled by a distance-dependent factor 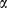 in Equation 2. It is clear, however, that the effect extends over tens to hundreds of meters which seem to be included in spatial working memory.

Another parameter in addition to the mere distance could be regionalization and spatial hierarchies. In virtual environments, navigators were shown to prefer routes that cross fewer region boundaries over equidistant routes through multiple regions [17]. In this experiment, regions were defined by the semantic class of landmark objects. In a pointing experiment, Wang and Brockmole [50] demonstrate that information from nested environments may be kept separate in spatial representations. In the city environments used in the present study, there are various configurations of buildings, roads, shops, etc. which segregate the environment into quarters, districts, neighborhoods, etc. Therefore it seems possible that the extension of spatial working memory is defined by region boundaries rather than by metric distance. This might also explain the results for the interview locations F and G in experiment 1 and C in experiment 2: They were probably attributed to the region “riverfront” and not “downtown”, and therefore no or only weak connections to the target places existed while the experiment took place.

The theoretical account presented in the Introduction is clearly able to explain our data. In addition, the findings by Basten et al. [21] on view-based priming of recall by mental travel also fit into the overall scheme. In this study, all interviews were carried out at a distant location (the North location in Fig. 3b) and simple recall of the “Holzmarkt” square revealed the same view preference reported here. Mental travel across the “Holzmarkt”, however, primed view-specific recall in the direction of travel, indicating that mental travel, just as actual walking in downtown Tübingen, activates view-specific working memories.

Alternative models of spatial working memory not based on views but on object representations and maps have been presented by [1], [2], [4]. While our data do not strictly rule out these models, they make clear that representations of places are not unique entities that are always activated in their entirety, but that parts of place representations can play independent roles in spatial recall. Such parts are oriented and have therefore been referred to as “views” in this paper. Alternatively, such parts could be landmarks or houses located at one side of a square, or names or other properties of such landmarks or houses, as might have been the case in the experiments reported by Bisiach and Luzzatti [22]. The considered parts of place representations are view-like in two respects: First, the target square effect (canonical view) shows that oriented parts of a place representation can be anisotropically distributed. Second, priming by spatial nearness activates oriented parts of the representation of places, not place representations in their entirety. This finding is in line with previous results of [41] who showed that associative landmark usage depends on oriented parts of place representations rather than on representations of entire places. Overall, we suggest that oriented “views” form a separate level of granularity in spatial representation that can be activated whenever view-specific information is required.

## Supporting Information

Figure S1
**Sketch orientation counts.** The figure shows the counts for the sketch orientations for all interview locations and both experiments as determined by the rating process. In rare cases, where all raters agreed that the orientation was between two cardinal directions (e.g., SW), a count of 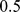 was added to each of the adjacent cardinal directns (e.g., S and W). The rightmost column of the table shows the airline directions from interview location to goal, computed according to Equation 1.(XLS)
